# 2-(4-Bromo­benzene­sulfonamido)-2-phenyl­acetic acid monohydrate

**DOI:** 10.1107/S160053680902830X

**Published:** 2009-07-22

**Authors:** Muhammad Nadeem Arshad, Islam Ullah Khan, Mehmet Akkurt, Muhammad Shafiq

**Affiliations:** a Department of Chemistry, Government College University, Lahore, Pakistan; b Department of Physics, Faculty of Arts and Sciences, Erciyes University, 38039 Kayseri, Turkey

## Abstract

In the title compound, C_14_H_12_BrNO_4_S·H_2_O, the phenyl and benzene rings are inclined at a dihedral angle of 39.5 (5)°. The crystal packing is stabilized by N—H⋯O, C—H⋯O and O—H⋯O hydrogen-bonding inter­actions.

## Related literature

For background to sulfonamide derivatives, see: Sheppard *et al.* (2006 ▶). For similar structures, see: Arshad *et al.* (2009 ▶); Asiri *et al.* (2009 ▶); Sethu Sankar *et al.* (2002 ▶); Wijeyesakere *et al.* (2008 ▶). For background to our study of the synthesis and structures of thia­zine-related heterocycles, see: Arshad *et al.* (2008 ▶). A related derivative has gained inter­est as a ligand in complex formation (Han *et al.*, 2006 ▶) and for its biological activity (Cama *et al.*, 2003 ▶; Dankwardt *et al.*, 2002 ▶). For the synthesis, see: Deng & Mani (2006 ▶). For bond-length data, see: Allen *et al.* (1987 ▶).
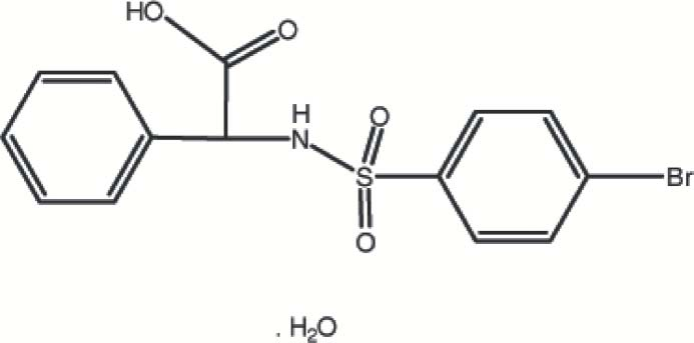


## Experimental

### 

#### Crystal data

C_14_H_12_BrNO_4_S·H_2_O
*M* *_r_* = 388.23
Orthorhombic, 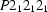

*a* = 5.5654 (13) Å
*b* = 16.230 (4) Å
*c* = 17.597 (4) Å
*V* = 1589.5 (6) Å^3^
*Z* = 4
Mo *K*α radiation
μ = 2.74 mm^−1^
*T* = 296 K
0.32 × 0.11 × 0.09 mm

#### Data collection

Bruker Kappa APEXII CCD area-detector diffractometer
Absorption correction: multi-scan (*SADABS*; Bruker 2007 ▶) *T*_min_ = 0.474, *T*_max_ = 0.791
9588 measured reflections
3315 independent reflections
1227 reflections with *I* > 2σ(*I*)
*R*_int_ = 0.083

#### Refinement

*R*[*F* ^2^ > 2σ(*F* ^2^)] = 0.057
*wR*(*F* ^2^) = 0.180
*S* = 0.93
3315 reflections
206 parameters
3 restraints
H atoms treated by a mixture of independent and constrained refinement
Δρ_max_ = 0.28 e Å^−3^
Δρ_min_ = −0.40 e Å^−3^
Absolute structure: Flack (1983 ▶), 1246 Freidel pairs
Flack parameter: 0.02 (3)

### 

Data collection: *APEX2* (Bruker, 2007 ▶); cell refinement: *SAINT* (Bruker, 2007 ▶); data reduction: *SAINT*; program(s) used to solve structure: *SIR97* (Altomare *et al.*, 1999 ▶); program(s) used to refine structure: *SHELXL97* (Sheldrick, 2008 ▶); molecular graphics: *ORTEP-3 for Windows* (Farrugia, 1997 ▶); software used to prepare material for publication: *WinGX* (Farrugia, 1999 ▶) and *PLATON* (Spek, 2009 ▶).

## Supplementary Material

Crystal structure: contains datablocks global, I. DOI: 10.1107/S160053680902830X/bt5013sup1.cif

Structure factors: contains datablocks I. DOI: 10.1107/S160053680902830X/bt5013Isup2.hkl

Additional supplementary materials:  crystallographic information; 3D view; checkCIF report

## Figures and Tables

**Table 1:** Hydrogen-bond geometry (Å, °)

*D*—H⋯*A*	*D*—H	H⋯*A*	*D*⋯*A*	*D*—H⋯*A*
N1—H1⋯O4^i^	0.86	2.47	3.113 (9)	132
O5—H*W*1⋯O1^ii^	0.85 (11)	2.56 (11)	3.027 (8)	116 (10)
O5—H*W*2⋯O3^iii^	0.85 (2)	2.34 (10)	2.956 (9)	131 (12)
O5—H*W*2⋯O2^iv^	0.85 (2)	2.28 (12)	2.868 (8)	127 (12)
O4—H4⋯O5	0.82	1.78	2.562 (8)	160
C6—H6⋯O2	0.93	2.51	2.897 (10)	105
C7—H7⋯O1^iii^	0.98	2.44	3.377 (10)	160
C7—H7⋯O2	0.98	2.48	2.958 (9)	109

Symmetry codes: (i) 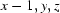; (ii) 
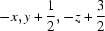
; (iii) 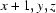; (iv) 
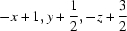
.

**Fig. 1.**
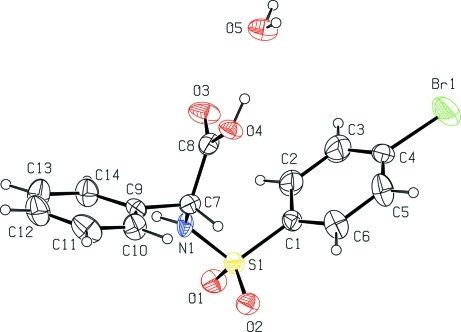
The molecular structure of (I), showing the atom labeling scheme and displacement ellipsoids are drawn at the 30% probability level.

**Fig. 2.**
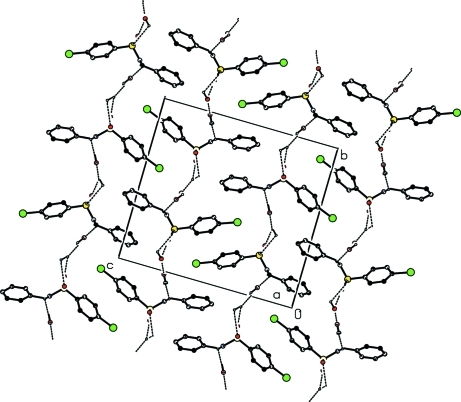
View of the crystal packing and hydrogen bonding (dashed lines) of the title compound, down the a-axis. H atoms not involved in the hydrogen bonding have been omitted for clarity.

## supplementary crystallographic information

### Comment

The title compound was synthesized *via* condensation reaction between 4-bromo benzene sulfonylchloride and phenylglycine, an amino acid. The related derivative has gained interest as a ligand in complexation (Han *et al.*, 2006) and biological activities (Dankwardt *et al.*, 2002, Cama *et al.*, 2003). It is the halogenated analogue of previously reported sulfonamide derivative of phenylglycine (Arshad *et al.*, 2009) in continuation of synthesis and crystal structure studies of thiazine related heterocycles (Arshad *et al.*, 2008).

In the title compound   (Fig. 1), the Br—C and C—O distances are as expected. The *S*—C_benzene_ distance of 1.754 (8) Å is shorter than the reported values of 1.763 (2)Å (Asiri *et al.*, 2009) and 1.763 (9) Å (Allen *et al.*, 1987). The S1—N1 distance of 1.617 (6) is shorter than the literature values of 1.6213 (18) Å (Asiri *et al.*, 2009) and 1.6458 (11) Å (Wijeyesakere *et al.*, 2008). The mean S=O distance of 1.439 (5) Å is comparable with the reported value of 1.436 (2) Å (Sethu Sankar *et al.*, 2002). The interplanar angle between the phenyl (C9–C14) and benzene (C1–C6) rings in (I) is 39.5 (5) °. These rings orient to the carbonyl group (C7/C8/O3/O4) with angles of 41.5 (5) and 77.1 (5) °, respectively.

The crystal packing is stabilized by N—H···O, C—H···O and O—H···O hydrogen bonding interactions (Table 1, Fig. 2).

### Experimental

The title compound was prepared in accordance with the literature method (Deng & Mani, 2006). Phenylglycine (1 g, 6.6 mmol) was dissolved in distilled water (10 ml) using 1*M*, Na_2_CO_3 _solution at pH 8–9. 4-Bromo benzene sulfonyl chloride (1.68 g, 6.6 mmol) was then added to the solution, which was stirred at room temperature until all the 4-bromobenzene sulfonyl chloride had been consumed. On completion of the reaction the pH was adjusted to 1–2, using 1 N HCl with stirring. The precipitate obtained was filtered, washed with distilled water, dried and recrystalized in methanol for X-ray diffraction studies.

### Refinement

The H atoms of the water molecule were found from a difference Fourier map and refined with distance restraints of O—H = 0.85 (1) Å and H···H = 1.39 (1) Å. The other H atoms were positioned geometrically and treated as riding, with C—H = 0.93–0.98 Å, N—H = 0.86 Å and O—H = 0.82 Å, with *U*_iso_(H) = 1.2*U*_eq_(C,*N*) and *U*_iso_(H) = 1.5*U*_eq_(O).

### Crystal data

**Table:**

C_14_H_12_BrNO_4_S·H_2_O	*F*(000) = 784
*M**_r_* = 388.23	*D*_x_ = 1.622 Mg m^−^^3^
Orthorhombic, *P*2_1_2_1_2_1_	Mo *K*α radiation, λ = 0.71073 Å
Hall symbol: P 2ac 2ab	Cell parameters from 868 reflections
*a* = 5.5654 (13) Å	θ = 2.3–16.4°
*b* = 16.230 (4) Å	µ = 2.74 mm^−^^1^
*c* = 17.597 (4) Å	*T* = 296 K
*V* = 1589.5 (6) Å^3^	Rod like, white
*Z* = 4	0.32 × 0.11 × 0.09 mm

### Data collection

**Table:**

Bruker Kappa APEXII CCD area-detector diffractometer	3315 independent reflections
Radiation source: sealed tube	1227 reflections with *I* > 2σ(*I*)
graphite	*R*_int_ = 0.083
φ and ω scans	θ_max_ = 27.2°, θ_min_ = 1.7°
Absorption correction: multi-scan (*SADABS*; Bruker 2007)	*h* = −7→6
*T*_min_ = 0.474, *T*_max_ = 0.791	*k* = −20→17
9588 measured reflections	*l* = −21→14

### Refinement

**Table:**

Refinement on *F*^2^	Secondary atom site location: difference Fourier map
Least-squares matrix: full	Hydrogen site location: inferred from neighbouring sites
*R*[*F*^2^ > 2σ(*F*^2^)] = 0.057	H atoms treated by a mixture of independent and constrained refinement
*wR*(*F*^2^) = 0.180	*w* = 1/[σ^2^(*F*_o_^2^) + (0.0737*P*)^2^] where *P* = (*F*_o_^2^ + 2*F*_c_^2^)/3
*S* = 0.93	(Δ/σ)_max_ < 0.001
3315 reflections	Δρ_max_ = 0.28 e Å^−^^3^
206 parameters	Δρ_min_ = −0.40 e Å^−^^3^
3 restraints	Absolute structure: Flack (1983), 1246 Freidel pairs
Primary atom site location: structure-invariant direct methods	Flack parameter: 0.02 (3)

### Special details

**Table:**

Geometry. Bond distances, angles *etc*. have been calculated using the rounded fractional coordinates. All su's are estimated from the variances of the (full) variance-covariance matrix. The cell e.s.d.'s are taken into account in the estimation of distances, angles and torsion angles
Refinement. Refinement on *F*^2^ for ALL reflections except those flagged by the user for potential systematic errors. Weighted *R*-factors *wR* and all goodnesses of fit *S* are based on *F*^2^, conventional *R*-factors *R* are based on *F*, with *F* set to zero for negative *F*^2^. The observed criterion of *F*^2^ > σ(*F*^2^) is used only for calculating -*R*-factor-obs *etc*. and is not relevant to the choice of reflections for refinement. *R*-factors based on *F*^2^ are statistically about twice as large as those based on *F*, and *R*-factors based on ALL data will be even larger.

### Fractional atomic coordinates and isotropic or equivalent isotropic displacement parameters (Å^2^)

**Table:**

	*x*	*y*	*z*	*U*_iso_*/*U*_eq_	
Br1	0.1150 (4)	0.40711 (9)	0.42252 (7)	0.1257 (7)	
S1	−0.1584 (4)	0.25772 (13)	0.74635 (12)	0.0429 (7)	
O1	−0.4134 (9)	0.2431 (3)	0.7477 (3)	0.054 (2)	
O2	0.0026 (9)	0.1905 (3)	0.7619 (3)	0.051 (2)	
O3	0.0588 (12)	0.4809 (4)	0.7664 (4)	0.068 (3)	
O4	0.4414 (11)	0.4403 (3)	0.7850 (3)	0.050 (2)	
N1	−0.1125 (12)	0.3278 (4)	0.8100 (3)	0.042 (3)	
C1	−0.0790 (16)	0.2999 (5)	0.6581 (4)	0.041 (3)	
C2	−0.228 (2)	0.3582 (6)	0.6266 (6)	0.073 (5)	
C3	−0.168 (3)	0.3924 (6)	0.5550 (6)	0.096 (6)	
C4	0.041 (3)	0.3666 (6)	0.5191 (5)	0.068 (5)	
C5	0.183 (2)	0.3106 (7)	0.5510 (5)	0.076 (5)	
C6	0.1220 (18)	0.2760 (6)	0.6224 (5)	0.065 (4)	
C7	0.1364 (15)	0.3510 (5)	0.8293 (4)	0.041 (3)	
C8	0.2012 (18)	0.4313 (6)	0.7896 (5)	0.047 (4)	
C9	0.1756 (17)	0.3609 (5)	0.9138 (4)	0.041 (3)	
C10	0.3725 (17)	0.3299 (5)	0.9486 (5)	0.057 (4)	
C11	0.412 (2)	0.3415 (7)	1.0241 (6)	0.074 (5)	
C12	0.250 (2)	0.3862 (7)	1.0669 (6)	0.073 (5)	
C13	0.046 (2)	0.4141 (7)	1.0329 (6)	0.073 (4)	
C14	0.0096 (17)	0.4023 (6)	0.9574 (5)	0.060 (4)	
O5	0.6162 (12)	0.5722 (4)	0.7272 (4)	0.064 (2)	
H1	−0.23170	0.35130	0.83230	0.0510*	
H2	−0.36690	0.37490	0.65180	0.0880*	
H3	−0.26630	0.43160	0.53250	0.1150*	
H4	0.47300	0.48030	0.75840	0.0750*	
H5	0.32320	0.29420	0.52640	0.0920*	
H6	0.22150	0.23670	0.64450	0.0770*	
H7	0.24430	0.30770	0.81070	0.0490*	
H10	0.48310	0.29990	0.92020	0.0680*	
H11	0.54870	0.31930	1.04690	0.0890*	
H12	0.27980	0.39700	1.11800	0.0870*	
H13	−0.06900	0.44150	1.06170	0.0880*	
H14	−0.12960	0.42250	0.93480	0.0720*	
HW1	0.556 (19)	0.606 (7)	0.696 (7)	0.1890*	
HW2	0.768 (2)	0.575 (9)	0.726 (7)	0.1890*	

### Atomic displacement parameters (Å^2^)

**Table:**

	*U*^11^	*U*^22^	*U*^33^	*U*^12^	*U*^13^	*U*^23^
Br1	0.2170 (18)	0.1032 (11)	0.0568 (7)	−0.0449 (12)	0.0187 (10)	0.0154 (7)
S1	0.0355 (11)	0.0463 (14)	0.0470 (13)	−0.0002 (12)	0.0066 (11)	0.0011 (12)
O1	0.028 (3)	0.058 (4)	0.076 (4)	−0.011 (3)	0.004 (3)	−0.002 (4)
O2	0.043 (3)	0.045 (4)	0.066 (4)	0.013 (3)	0.008 (3)	0.005 (3)
O3	0.049 (4)	0.055 (4)	0.101 (5)	0.011 (3)	−0.003 (4)	0.028 (4)
O4	0.043 (4)	0.043 (4)	0.065 (4)	−0.003 (3)	0.013 (3)	0.014 (3)
N1	0.026 (4)	0.059 (5)	0.042 (4)	−0.001 (4)	0.013 (3)	−0.014 (3)
C1	0.035 (6)	0.045 (6)	0.043 (5)	−0.003 (5)	0.004 (4)	−0.001 (4)
C2	0.101 (10)	0.060 (7)	0.059 (7)	0.034 (7)	0.007 (6)	0.007 (6)
C3	0.143 (13)	0.068 (8)	0.077 (9)	0.033 (9)	0.018 (9)	0.005 (7)
C4	0.122 (11)	0.042 (6)	0.041 (6)	−0.020 (7)	0.005 (7)	0.001 (5)
C5	0.063 (8)	0.111 (10)	0.054 (7)	0.009 (7)	0.015 (6)	0.005 (7)
C6	0.052 (7)	0.089 (8)	0.053 (6)	−0.007 (6)	−0.002 (5)	0.005 (6)
C7	0.030 (5)	0.039 (5)	0.055 (6)	−0.002 (4)	0.006 (4)	−0.003 (4)
C8	0.038 (7)	0.046 (7)	0.056 (6)	−0.010 (5)	0.011 (5)	−0.014 (5)
C9	0.043 (6)	0.042 (5)	0.037 (5)	−0.008 (5)	0.000 (5)	0.008 (4)
C10	0.044 (7)	0.068 (7)	0.059 (7)	0.021 (5)	0.003 (5)	0.007 (5)
C11	0.058 (8)	0.091 (8)	0.073 (8)	0.007 (7)	−0.015 (7)	0.034 (7)
C12	0.079 (9)	0.098 (10)	0.042 (6)	0.010 (6)	−0.010 (6)	0.014 (6)
C13	0.076 (8)	0.089 (8)	0.054 (7)	0.017 (7)	−0.002 (6)	−0.013 (6)
C14	0.057 (7)	0.077 (8)	0.045 (6)	0.012 (6)	−0.011 (5)	0.003 (6)
O5	0.055 (4)	0.054 (4)	0.083 (4)	−0.010 (3)	−0.007 (4)	0.014 (3)

### Geometric parameters (Å, °)

**Table:**

Br1—C4	1.868 (10)	C7—C8	1.522 (12)
S1—O1	1.439 (5)	C7—C9	1.511 (10)
S1—O2	1.438 (5)	C9—C14	1.376 (13)
S1—N1	1.617 (6)	C9—C10	1.352 (13)
S1—C1	1.754 (8)	C10—C11	1.360 (14)
O3—C8	1.201 (12)	C11—C12	1.381 (16)
O4—C8	1.347 (12)	C12—C13	1.361 (16)
O4—H4	0.8200	C13—C14	1.358 (14)
O5—HW1	0.85 (11)	C2—H2	0.9300
O5—HW2	0.85 (2)	C3—H3	0.9300
N1—C7	1.475 (11)	C5—H5	0.9300
N1—H1	0.8600	C6—H6	0.9300
C1—C2	1.375 (13)	C7—H7	0.9800
C1—C6	1.340 (13)	C10—H10	0.9300
C2—C3	1.417 (15)	C11—H11	0.9300
C3—C4	1.39 (2)	C12—H12	0.9300
C4—C5	1.329 (17)	C13—H13	0.9300
C5—C6	1.418 (13)	C14—H14	0.9300
			
Br1···O4^i^	3.477 (5)	C13···H3^xii^	2.9500
Br1···C8^i^	3.660 (10)	C13···HW1^v^	2.94 (12)
O1···C7^ii^	3.377 (10)	C14···H1	2.7100
O1···O5^iii^	3.027 (8)	H1···H14	2.2200
O2···O5^iv^	2.868 (8)	H1···H10^ii^	2.3700
O3···N1	2.770 (9)	H1···O4^ii^	2.4700
O3···O5^ii^	2.956 (9)	H1···C10^ii^	3.0300
O4···O5	2.562 (8)	H1···O3	2.9000
O4···C10	3.413 (10)	H1···C14	2.7100
O4···Br1^v^	3.477 (5)	HW1···C13^i^	2.94 (12)
O4···N1^vi^	3.113 (9)	HW1···H12^i^	2.3200
O5···O3^vi^	2.956 (9)	HW1···C12^i^	2.84 (12)
O5···O2^vii^	2.868 (8)	HW1···H4	2.3600
O5···O1^viii^	3.027 (8)	HW1···O2^vii^	2.91 (11)
O5···O4	2.562 (8)	HW1···H13^i^	2.4900
O1···HW1^iii^	2.56 (11)	HW1···O1^viii^	2.56 (11)
O1···H6^ii^	2.7300	H2···O1	2.7400
O1···H7^ii^	2.4400	H2···O4^ii^	2.7900
O1···H2	2.7400	HW2···O3^vi^	2.34 (10)
O2···H7	2.4800	HW2···O2^vii^	2.28 (12)
O2···H6	2.5100	HW2···H4	2.3200
O2···HW1^iv^	2.91 (11)	H3···H13^x^	2.3100
O2···H12^ix^	2.8300	H3···C13^x^	2.9500
O2···HW2^iv^	2.28 (12)	H4···HW2	2.3200
O3···HW2^ii^	2.34 (10)	H4···O5	1.7800
O3···H1	2.9000	H4···HW1	2.3600
O4···H2^vi^	2.7900	H5···C5^xiii^	2.9600
O4···H1^vi^	2.4700	H5···C4^xiii^	2.9900
O5···H4	1.7800	H6···O1^vi^	2.7300
N1···O4^ii^	3.113 (9)	H6···O2	2.5100
N1···O3	2.770 (9)	H7···O1^vi^	2.4400
N1···H14	2.6800	H7···O2	2.4800
C1···C8	3.512 (12)	H7···H10	2.3400
C7···O1^vi^	3.377 (10)	H10···H1^vi^	2.3700
C8···Br1^v^	3.660 (10)	H10···H7	2.3400
C8···C1	3.512 (12)	H11···C9^xiv^	3.0900
C10···O4	3.413 (10)	H11···C10^xiv^	3.0200
C3···H13^x^	3.0700	H12···HW1^v^	2.3200
C4···H5^xi^	2.9900	H12···O2^xiv^	2.8300
C5···H5^xi^	2.9600	H13···C3^xii^	3.0700
C9···H11^ix^	3.0900	H13···HW1^v^	2.4900
C10···H1^vi^	3.0300	H13···H3^xii^	2.3100
C10···H11^ix^	3.0200	H14···N1	2.6800
C12···HW1^v^	2.84 (12)	H14···H1	2.2200
			
O1—S1—O2	119.1 (3)	C7—C9—C14	120.2 (8)
O1—S1—N1	105.1 (3)	C10—C9—C14	118.2 (7)
O1—S1—C1	109.1 (4)	C9—C10—C11	121.5 (9)
O2—S1—N1	107.7 (3)	C10—C11—C12	120.0 (10)
O2—S1—C1	107.9 (4)	C11—C12—C13	118.7 (10)
N1—S1—C1	107.4 (4)	C12—C13—C14	120.5 (10)
C8—O4—H4	109.00	C9—C14—C13	121.0 (9)
HW1—O5—HW2	110 (12)	C3—C2—H2	120.00
S1—N1—C7	119.2 (5)	C1—C2—H2	121.00
C7—N1—H1	120.00	C2—C3—H3	121.00
S1—N1—H1	120.00	C4—C3—H3	120.00
S1—C1—C6	120.8 (7)	C4—C5—H5	120.00
S1—C1—C2	118.3 (7)	C6—C5—H5	120.00
C2—C1—C6	120.9 (8)	C5—C6—H6	120.00
C1—C2—C3	119.1 (10)	C1—C6—H6	120.00
C2—C3—C4	119.0 (11)	C8—C7—H7	108.00
Br1—C4—C5	119.6 (10)	C9—C7—H7	108.00
C3—C4—C5	120.8 (9)	N1—C7—H7	108.00
Br1—C4—C3	119.5 (9)	C11—C10—H10	119.00
C4—C5—C6	120.2 (10)	C9—C10—H10	119.00
C1—C6—C5	120.1 (9)	C10—C11—H11	120.00
N1—C7—C9	112.9 (6)	C12—C11—H11	120.00
C8—C7—C9	109.1 (7)	C13—C12—H12	121.00
N1—C7—C8	109.6 (7)	C11—C12—H12	121.00
O3—C8—O4	124.2 (9)	C12—C13—H13	120.00
O3—C8—C7	125.0 (9)	C14—C13—H13	120.00
O4—C8—C7	110.8 (7)	C9—C14—H14	120.00
C7—C9—C10	121.5 (8)	C13—C14—H14	120.00
			
O1—S1—N1—C7	173.4 (5)	C3—C4—C5—C6	0.3 (17)
O2—S1—N1—C7	45.5 (6)	C4—C5—C6—C1	−0.2 (16)
C1—S1—N1—C7	−70.5 (6)	N1—C7—C8—O3	21.3 (12)
O1—S1—C1—C2	40.8 (8)	N1—C7—C8—O4	−159.8 (6)
O2—S1—C1—C2	171.6 (7)	C9—C7—C8—O3	−102.8 (10)
N1—S1—C1—C2	−72.6 (8)	C9—C7—C8—O4	76.1 (9)
O1—S1—C1—C6	−139.3 (7)	N1—C7—C9—C10	136.2 (8)
O2—S1—C1—C6	−8.6 (8)	N1—C7—C9—C14	−43.9 (11)
N1—S1—C1—C6	107.3 (8)	C8—C7—C9—C10	−101.7 (9)
S1—N1—C7—C8	101.1 (7)	C8—C7—C9—C14	78.2 (10)
S1—N1—C7—C9	−137.1 (6)	C7—C9—C10—C11	177.5 (9)
S1—C1—C6—C5	179.8 (7)	C14—C9—C10—C11	−2.4 (14)
S1—C1—C2—C3	−179.4 (8)	C7—C9—C14—C13	−177.8 (9)
C6—C1—C2—C3	0.7 (15)	C10—C9—C14—C13	2.1 (14)
C2—C1—C6—C5	−0.3 (14)	C9—C10—C11—C12	−0.4 (16)
C1—C2—C3—C4	−0.7 (16)	C10—C11—C12—C13	3.5 (17)
C2—C3—C4—C5	0.2 (18)	C11—C12—C13—C14	−3.8 (17)
C2—C3—C4—Br1	177.0 (8)	C12—C13—C14—C9	1.0 (16)
Br1—C4—C5—C6	−176.5 (8)		

Symmetry codes: (i) −*x*+1/2, −*y*+1, *z*−1/2; (ii) *x*−1, *y*, *z*; (iii) −*x*, *y*−1/2, −*z*+3/2; (iv) −*x*+1, *y*−1/2, −*z*+3/2; (v) −*x*+1/2, −*y*+1, *z*+1/2; (vi) *x*+1, *y*, *z*; (vii) −*x*+1, *y*+1/2, −*z*+3/2; (viii) −*x*, *y*+1/2, −*z*+3/2; (ix) *x*−1/2, −*y*+1/2, −*z*+2; (x) −*x*−1/2, −*y*+1, *z*−1/2; (xi) *x*−1/2, −*y*+1/2, −*z*+1; (xii) −*x*−1/2, −*y*+1, *z*+1/2; (xiii) *x*+1/2, −*y*+1/2, −*z*+1; (xiv) *x*+1/2, −*y*+1/2, −*z*+2.

### Hydrogen-bond geometry (Å, °)

**Table:**

*D*—H···*A*	*D*—H	H···*A*	*D*···*A*	*D*—H···*A*
N1—H1···O4^ii^	0.86	2.47	3.113 (9)	132
O5—HW1···O1^viii^	0.85 (11)	2.56 (11)	3.027 (8)	116 (10)
O5—HW2···O3^vi^	0.85 (2)	2.34 (10)	2.956 (9)	131 (12)
O5—HW2···O2^vii^	0.85 (2)	2.28 (12)	2.868 (8)	127 (12)
O4—H4···O5	0.82	1.78	2.562 (8)	160
C6—H6···O2	0.93	2.51	2.897 (10)	105
C7—H7···O1^vi^	0.98	2.44	3.377 (10)	160
C7—H7···O2	0.98	2.48	2.958 (9)	109

Symmetry codes: (ii) *x*−1, *y*, *z*; (viii) −*x*, *y*+1/2, −*z*+3/2; (vi) *x*+1, *y*, *z*; (vii) −*x*+1, *y*+1/2, −*z*+3/2.
